# Integrated PM–MOX–Thermal Sensing for Monitoring Bioaerosol Dynamics in Controlled Indoor Environments

**DOI:** 10.3390/s26113521

**Published:** 2026-06-02

**Authors:** Maria Inês Barbosa, Hugo Roxo, Pedro Ribeiro, José Menezes, Eduarda Vieira, Patrícia Moreira, Pedro Miguel Rodrigues

**Affiliations:** 1 CBQF—Centro de Biotecnologia e Química Fina—Laboratório Associado, Escola Superior de Biotecnologia, Universidade Católica Portuguesa, Rua de Diogo Botelho 1327, 4169-005 Porto, Portugal; mibarbosa@ucp.pt (M.I.B.); s-hroxo@ucp.pt (H.R.); s-pmsbribeiro@ucp.pt (P.R.); s-jmmenezes@ucp.pt (J.M.); 2 CITAR—Centro de Investigação em Ciência e Tecnologia das Artes—Escola das Artes, Universidade Católica Portuguesa, Rua de Diogo Botelho 1327, 4169-005 Porto, Portugal; evieira@ucp.pt (E.V.); prmoreira@ucp.pt (P.M.)

**Keywords:** bioaerosols, indoor contamination monitoring, multisensor systems, MOX, mVOCs, particulate matter sensing, thermographic sensing, *Penicillium chrysogenum*

## Abstract

Indoor monitoring of biological contamination is essential for protecting cultural heritage and public health. However, conventional culture-based methods limit timely intervention. This study presents an affordable modular multisensor system for indirectly detecting airborne fungal contamination using *Penicillium chrysogenum* as a representative model organism and its environmental signatures. The proposed prototype integrates PMSA003I, BME688 and AMG8833 sensors and was evaluated under controlled environmental conditions. Biological ground truth was established using a volumetric inertial-impaction sampling protocol (SAS sampler), validating four contamination levels (~6 to 165, CFU/m^3^). A total of 1989 observations were analyzed. Non-parametric statistical tests (Kruskal–Wallis and Mann–Whitney *U*) confirmed significant differences between all the exposure conditions (p<0.001). Supervised machine learning (ML) models showed strong performance across all the classification tasks, with *accuracy* and *AUC* values near 100%. In most cases, pressure alone was sufficient. The statistical and ML analyses consistently identified pressure, particulate-related variables, gas resistance and humidity as the most informative features. Overall, the results indicate that the proposed approach can reliably capture indirect environmental signatures associated with airborne fungal presence under controlled conditions. The study supports the feasibility of low-cost multisensor systems for continuous indoor bioaerosol monitoring while highlighting the need for further optimization and validation in real-world environments.

## 1. Introduction

In recent decades, the profound impact of environmental factors, particularly air quality, on human health has gained widespread recognition. This issue is especially critical in occupational settings, where workers may experience prolonged exposure to hazardous pollutants. Accurate and reliable air quality monitoring is therefore essential for safeguarding health, improving life expectancy and informing regulatory actions. Growing evidence links poor air quality not only to acute respiratory and cardiovascular morbidity but also to chronic diseases, cancer, cognitive decline and increased all-cause mortality [1,2]. Specifically, biological particulate matter (PM) or bioaerosols with an aerodynamic diameter of <5 μm represent a unique threat. These particles can bypass mucociliary clearance mechanisms, deposit in the alveoli and trigger distinct immunopathological responses. The World Health Organization (WHO) attributes more than 7 million premature deaths globally each year to air pollution, a huge proportion of which are associated with occupational or indoor exposure [3].

Workplaces frequently contain high concentrations of hazardous pollutants, including carcinogenic agents (e.g., asbestos and diesel engine exhaust), PM_2.5_ and volatile organic compounds (VOCs). Chronic exposure to these pollutants drives systemic inflammation, respiratory and cardiovascular disease, asthma exacerbation and increased disability-adjusted life years [1,4]. Instruments used to measure PM mainly focus on particle size and concentration and are based on methods such as particle charging, size classification, and detection using optical or electrical sensors, as well as gravimetric and microbalance techniques. However, these methods have limitations, including trade-offs between accuracy and time resolution, sensitivity to environmental conditions, reliance on calibration assumptions and, in some cases, high cost and complexity. Therefore, a combination of methods is often required to obtain more reliable results [5,6]. VOCs are most commonly measured using gas chromatography, where the compounds are separated, typically on nonpolar columns, and then detected using instruments such as flame ionization detectors, electron capture detectors or, most often, mass spectrometers [7], which provide high sensitivity and specificity but require expensive instrumentation, complex sample preparation and are not suitable for continuous real-time monitoring [8,9]. While conventional techniques usually offer high accuracy and specificity, their limited temporal resolution, cost and operational complexity restrict their use for continuous monitoring. In contrast, cloud-connected sensor networks enable real-time monitoring and threshold-based interventions, supporting timely warnings and safety measures in high-risk environments [4,10].

Pollution risks also extend to non-industrial indoor environments, where dust and fungal bioaerosols, commonly from *Aspergillus*, *Penicillium* and *Cladosporium*, represent significant health threats. Their small aerodynamic size (<5 μm) allows deep lung deposition, triggering allergic, inflammatory and invasive diseases, with invasive aspergillosis alone causing over 300,000 cases annually and high mortality in vulnerable populations [11,12,13]. Libraries, museums and archives are environments of particular concern for both human health and cultural heritage preservation. While serving as custodians of valuable collections, these spaces often exhibit micro-climatic conditions that are favorable to microbial growth, including poor ventilation, elevated relative humidity (>60%) and increased CO_2_ levels (>1000 ppm), which promote spore suspension and surface colonization [11,14]. Airborne microbial concentrations exceeding 500 CFU/m^3^ for fungi and 300 CFU/m^3^ for bacteria present dual risks by compromising human health and accelerating the deterioration of sensitive organic materials, such as paper, textiles and leather [15,16]. Among the fungi commonly detected in heritage environments, *Penicillium chrysogenum* represents a significant biological risk due to its production of easily aerosolized conidia that persist in indoor dust. Inhalation of these spores is associated with allergic responses, asthma and respiratory irritation in visitors and conservation staff. From a conservation perspective, *Penicillium chrysogenum* exhibits strong enzymatic activity that produces metabolic byproducts that accelerate cellulose hydrolysis and cause characteristic yellow staining of paper and book bindings, compromising material integrity [17]. The impact on indoor air quality and collection preservation highlights the need for targeted microbial monitoring within preventive conservation strategies for museum microclimates [18].

Air quality monitoring emerged during the Industrial Revolution, when pollution-related mortality underscored the need for systematic surveillance. Early methods relied on visual assessments, such as the Ringelmann chart, for smoke density, or on soot collection gauges [19,20]. By the mid-20th century, automated gas analyzers and PM samplers enabled continuous measurement and supported the development of emerging regulatory frameworks. Microprocessor-based devices introduced during the 1980s and 1990s further expanded monitoring capabilities, supporting large-scale real-time monitoring networks. More recently, low-cost portable sensors based on metal–oxide semiconductors, electrochemical cells and optical particle counters have democratized air quality monitoring [21], enabling a greater number and diversity of monitoring devices. Despite these advances, challenges persist, including calibration requirements, long-term sensor drift and stability issues, as well as ongoing concerns regarding measurement reliability [22,23].

Conventional approaches for fungal detection rely on laboratory-based methods, such as culturing techniques and morphological/colony-based characterization. However, these methods are often slow and require significant effort, taking several days to weeks to obtain results. They may also miss non-culturable or non-viable spores, resulting in an underestimation of total fungal spore counts despite their potential to cause allergies and health issues [24]. A Hirst-type volumetric sampler combined with light microscopic analysis is also widely used and considered reliable in aerobiology. However, it is also slow and requires considerable effort, with results often available only after days or weeks. In addition, variations in airflow conditions, counting procedures and operator experience can affect measurement accuracy [25]. To address these limitations, recent research has focused on indirect detection approaches based on environmental sensing, where fungal presence is inferred from characteristic signatures, such as PM, microbial VOCs, temperature, humidity and thermal anomalies, rather than direct spore counting [26,27]. Multisensor systems integrating low-cost particulate sensors and metal–oxide semiconductor gas sensors have shown promising results in identifying mold-prone indoor environments. In parallel, machine learning (ML) techniques applied to sensor data have significantly improved detection performance. For example, Borowik et al. demonstrated indirect rapid identification of fungal species without relying on traditional spore-based methods by using low-cost electronic noses based on metal–oxide (MOX) gas sensors and ML [28]. Choi et al. detected indoor fungal contamination by identifying microbial VOCs using SPME-GC–MS analysis, where VOC emission patterns were classified using ML models, achieving 96.2% *accuracy* based on key compounds such as styrene, ethanol and benzene [29]. More recently, deep learning-based frameworks using VOC sensor arrays composed of cross-sensitive MOX sensors, electrochemical gas sensors and environmental sensors (temperature and humidity) have demonstrated up to 89.9% *accuracy* in fungal species identification and approximately 87% *accuracy* in source localization [30]. Despite these high *accuracy* values, most ML-based systems rely on a single sensing modality and lack direct microbiological validation, meaning that performance metrics may not fully reflect the actual airborne fungal load. This highlights the need for affordable modular multisensor platforms that combine complementary environmental indicators with biological ground truth, enabling robust and interpretable monitoring of indoor fungal contamination.

The objective of this work is to develop and validate (via statistical tests and ML algorithms) an affordable multisensor system that is capable of detecting the presence of airborne spores through indirect environmental signatures. To achieve this, the study:

- Develops a modular open-source multisensor prototype that integrates PM, MOX and thermodynamic sensors into a compact low-cost platform designed for real-time monitoring of environmental features associated with airborne fungal spore presence under controlled and semi-controlled conditions.
- Designs the overall system architecture of the multisensor platform, defining the hardware integration strategy, sensor configuration, data acquisition pipeline and communication framework.
- Implements a controlled environmental-chamber protocol to generate reproducible bioaerosol dispersion events using *Penicillium chrysogenum* as the biological target, a species widely documented in indoor heritage environments.
- Establishes robust biological ground-truth data through volumetric impaction sampling, generating a high-quality accurately labeled dataset of fungal spore presence levels (CFU/m^3^) that supports validation, statistical inference and performance assessment supported by ML tools.
- Applies rigorous statistical methodologies to quantify detection performance across physical and chemical sensing domains and to identify the specific variables that constitute the most informative indirect markers of airborne fungal spore presence.

## 2. Methods

### 2.1. Microorganism Inoculation Protocol

Cultures of *Penicillium chrysogenum* were used to generate controlled fungal growth and spore loads for experimental validation. This species was selected due to its high prevalence in indoor and heritage environments and its suitability for laboratory handling under reduced biosafety constraints [31,32,33]. Owing to its small (2–4 µm) and readily aerosolized spores, as well as its release of oxidizing microbial VOCs, *Penicillium chrysogenum* provides stable and distinctive physicochemical signatures, thereby constituting an appropriate and robust model organism for benchmarking multisensor detection performance.

A local airborne isolate was obtained by passive air sampling in *Universidade Católica Portuguesa* (UCP) indoor spaces (41°09′11.57″ N, 8°40′17.00″ W) and purified by subculturing to ensure culture uniformity. Species identification was confirmed by macroscopic colony morphology and microscopic examination of characteristic conidiophores and conidia.

Four Petri dishes containing Potato Dextrose Agar (PDA, Difco, Detroit, MI, USA) were prepared under sterile conditions. Each plate was inoculated centrally using a sterile inoculation needle carrying conidia from the same parental culture to minimize inoculum variability. Cultures were incubated at 25 °C under static conditions and ambient laboratory humidity. To obtain reproducible differences in colony development and spore production, plates were incubated for two predefined durations: 2 days, corresponding to early growth, and 5 days, corresponding to mature sporulation. This enabled controlled modulation of colony size and spore density for subsequent aerosolization experiments.

### 2.2. Prototype

The experimental prototype is a modular embedded sensing platform that integrates multiple commercial sensors to detect and characterize bioaerosol events. Its architecture is organized into four functional layers: the *perception layer*, which performs environmental sensing and real-time data acquisition; the *communication*/*network layer*, responsible for transmitting data between the embedded device and the user interface; the *application layer*, which handles visualization, system control and user interaction; and the *data layer*, dedicated to structured storage and management of the acquired measurements. The following subsections describe each layer in detail.

#### 2.2.1. *Perception Layer*/Hardware

The prototype integrates a heterogeneous suite of sensors to enable indirect detection of airborne fungal spores through complementary physical and chemical signatures. PM (PM1.0, PM2.5, and ${\mathrm{PM}}_{10}$) and particle count (PC) (>0.3, >0.5, >1.0 and >2.5, μm) were measured using a Plantower PMSA0031 optical particle counter based on laser-scattering principles. Thermal information was acquired using a Panasonic AMG8833 infrared thermopile array, consisting of an 8×8 grid of sensing elements providing a spatial temperature map of the chamber. This was included to support real-time visual monitoring of spatial temperature distribution within the system as an extra capability. Environmental parameters, including ambient temperature, relative humidity, barometric pressure and metal–oxide gas resistance, were measured using the Bosch Sensortec BME680/BME688 family of sensors. Additional gas-related indicators (nitrogen dioxide (NO_2_) and ozone (O_3_)) and aggregated air-quality index (AQI)) were obtained using the Nicla Sense ME platform, integrating Bosch and Renesas components. Table 1 summarizes the main detection characteristics and metrological performance of the sensors used in the experimental setup, detailing the measured variables, sensing principles, associated resolution and performance specifications.

**Figure 1:**
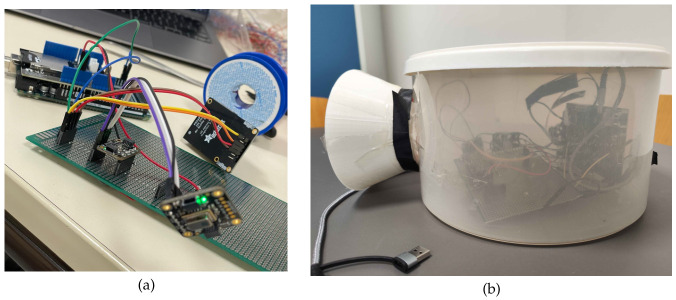
Final version of the electronic assembly (**a**) before and (**b**) after enclosure.

All sensors interface with an Arduino Portenta H7 microcontroller (Figure 1), which acts as the central coordination unit for synchronized data acquisition and overall device management. The sensing assembly is enclosed in an acrylic chamber to isolate the instrumentation from ambient conditions and to enable controlled exposure to fungal aerosols.

The firmware, implemented in C++ using the Arduino IDE, relies on vendor-supplied libraries and standard communication protocols (I^2^C, SPI, UART and GPIO) to ensure reliable interaction with all sensing modules. To maintain consistent sampling across heterogeneous sensors, the acquisition process is governed by a non-blocking timing loop: a millisecond counter triggers each polling cycle every 2 s, providing deterministic and repeatable sampling intervals while allowing concurrent device operations to run uninterrupted. This timing strategy ensures stable synchronization between sensors and prevents conflicts between acquisition, processing and communication tasks.

##### The Box (Acrylic Chamber) and Sensors’ Positions

**Figure 2:**
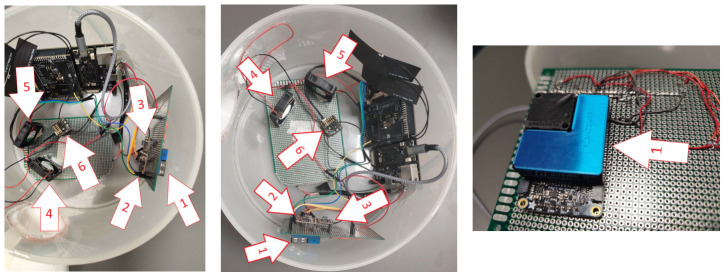
Sensor placement inside the acrylic chamber: (1) PMSA003I sensor; (2) AMG8833 IR 8 × 8 sensor; (3) BME680 environmental sensor; (4) and (5) ventilation inlets; (6) Arduino Nicla Sense Env sensor.

As illustrated in Figure 2, the first sensor (PMSA003I) is positioned below the remaining sensors to take advantage of the airflow generated by the ventilation system (vents 4 and 5), which directs air towards this location. This placement promotes the efficient transport of airborne particles to the sensing region, thereby enhancing particle detection efficiency and overall measurement accuracy.

The second (AMG8833 IR 8×8) and third (BME680) sensors are oriented upwards as the air inside the acrylic chamber is assumed to be homogeneous, ensuring representative and reliable measurements. Sensor 6, the Arduino Nicla Sense Env, is installed in close proximity to sensors 2 and 3 for the same reason. However, due to its operational characteristics, this sensor allows greater flexibility in positioning since the ventilation system does not introduce adverse effects on its measurements.

#### 2.2.2. Communication/Network Layer

Communication between components is established through standard digital buses, primarily I^2^C (Inter-Integrated Circuit) and UART, and synchronized within a unified acquisition cycle to ensure reliable transmission of sensor data from the embedded platform to both the real-time visualization interface and the cloud-based persistence system. The architecture follows a bidirectional edge-to-client-to-cloud model, separating local device communication from internet-based storage.

At the device level, the embedded controller transmits structured sensor records via USB serial communication. Data packets are encoded in JSON format to ensure platform independence and efficient parsing. Each acquisition cycle generates a complete record containing raw sensor measurements and derived features, which is serialized and streamed through the serial interface. On the client side, a browser-based dashboard built with React/Vite environment (React 19.2, Vite 7.0) communicates directly with the embedded system using the Web Serial API, enabling microcontroller-to-browser communication without additional middleware. Upon receiving each JSON payload, the client validates its structure, computes supplementary descriptors when necessary (e.g., condensed thermal matrix features) and prepares the data for cloud submission.

Cloud persistence is managed using the Google Firebase Realtime Database, a NoSQL JSON-tree service optimized for low-latency synchronization. Data are transmitted from the client to the backend via HTTPS using RESTful POST requests. Each record is stored under a timestamped hierarchical path (sensor_data/YYYY-MM-DD/hh-mm-ss), ensuring chronological traceability and preventing key collisions. Authentication and write permissions are enforced through Google Firebase security rules and API key validation, restricting unauthorized access to the database.

#### 2.2.3. Application Layer (Front End)

**Figure 3:**
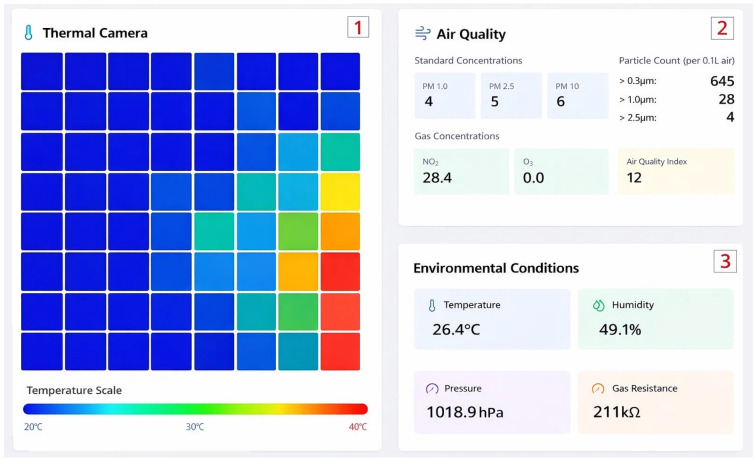
User interface of the developed monitoring system displaying real-time data acquired from the sensing platform. The interface is organized into three visualization main blocks: (1) thermal camera map, (2) air quality measurements and (3) environmental conditions.

The front-end dashboard, developed with React/Vite, serves as the primary interface for real-time visualization of sensor measurements (Figure 3). During each acquisition cycle, data are collected by the Arduino-based embedded system and transmitted to the host computer. Each acquisition cycle produces a structured data record containing the thermal matrix, PM concentrations, gas indicators and environmental parameters, which is formatted into a JSON object. This JSON-formatted data is subsequently distributed both to the dataset storage system for persistence and to the dashboard via the Web Serial API (communication/network layer). The dashboard renders raw and derived visual representations to support immediate inspection and validation of environmental conditions. Operating exclusively at the application level, this layer focuses on visualization and system control while complementing the cloud storage component responsible for the persistent archival of raw sensor streams.

The interface shown in Figure 3 is organized into three main visualization blocks. The first block corresponds to the thermal imaging module, where the infrared sensor output is rendered as a pseudo-color temperature map, providing a real-time 8×8 spatial distribution of surface temperatures inside the chamber.

The second block aggregates the air-quality indicators. This panel displays both mass concentrations of PM (PM_1.0_, PM_2.5_ and PM_10.0_, expressed in µg/m^3^) and PCs distributed across predefined aerodynamic size bins (>0.3 µm, >1.0 µm, and >2.5 µm). It also includes gas-sensor responses and derived chemical indicators (NO_2_, O_3_ and AQI). Together, these variables offer a high-resolution characterization of the aerosol and chemical composition of the monitored environment.

The third block presents the environmental parameters, including ambient temperature (°C), relative humidity (%), barometric pressure (hPa) and metal--oxide gas resistance (kΩ), reflecting the thermodynamic and humidity conditions that modulate aerosol behavior. All numerical values displayed in the interface correspond to real-time measurements acquired by the sensing platform at the embedded sampling rate.

#### 2.2.4. Data Layer

The data layer ensures the structured, persistent and traceable storage of all measurements generated by the sensing platform. Each acquisition cycle produces a timestamped JSON record that encapsulates the complete set of raw outputs from all sensing modules and is archived without modification through the cloud persistence backend/data layer.

The stored dataset reflects the multimodal nature of the sensing architecture: each record includes 15 time-series variables, covering particulate concentrations, gas indicators, environmental conditions and the 8 × 8 thermal matrix. Together, these complementary data channels provide a structured and multidimensional representation of the chamber’s environmental state at each sampling instant.

Data are organized in a hierarchical chronologically ordered structure that preserves the original sampling resolution and all associated metadata. This organization supports efficient querying, reconstruction of temporal profiles and seamless integration with downstream analytical pipelines. By maintaining the integrity and provenance of each record, the data layer guarantees full reproducibility across the entire acquisition pipeline.

### 2.3. Prototype Validation

Data were collected under controlled experimental conditions to evaluate and validate sensor responses associated with distinct levels of microbial presence. This validation stage was fundamental not only to characterize the behavior of each individual sensor but also to assess the performance of the complete ensemble within an integrated *all-in-box* solution. In this work, *Penicillium chrysogenum* was used as a model organism to represent airborne fungal contamination. A custom prototype was employed to suspend spores and simultaneously record environmental parameters relevant to bioaerosol detection.

**Figure 4:**
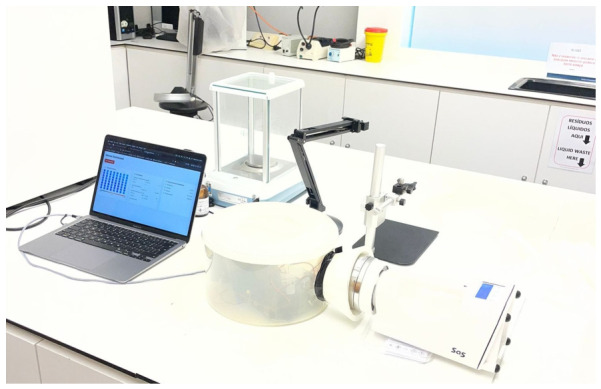
Experimental validation setup. The acrylic chamber isolates the sensor array and the biological culture. The integrated fan system creates a turbulent flow, forcing the aerosolization of spores.

- **Experimental Setup and Aerosolization**: All experiments were conducted within a sealed acrylic chamber housing the sensor array, data acquisition unit and low-power ventilators. The ventilators were activated during measurements to generate homogeneous airflow and promote spore suspension, simulating indoor air disturbances. A 3D-printed PLA support structure guided aerosolized particles toward a Petri dish positioned within a Surface Air System (SAS) sampler, VWR International, USA. Active aerosolization was achieved by operating internal fans during data acquisition, inducing turbulent airflow that detached and suspended fungal conidia from culture plates. Concurrently, bioaerosols were quantified by optical PM sensors and physically collected via inertial impaction using the SAS sampler (total sampled volume: 1 m^3^). The experimental validation setup is illustrated in Figure 4.
- **Sample Collection and Synchronization**: After each SAS sampling cycle, the inoculated Petri dishes were aseptically retrieved and incubated under predefined culture conditions. Following incubation, colony-forming units (CFUs) were enumerated and normalized to the sampled air volume, with results expressed as CFU/m^3^. These values represent an estimate of the concentration of culturable airborne fungal spores at each sampling point [37]. Each sample was uniquely labeled to ensure full traceability and synchronization with the corresponding sensor data (i.e., recorded start and end times of the SAS acquisition interval). Since each SAS sample provides a CFU value integrated over the entire time required to collect 1 m^3^ of air, all high-frequency sensor measurements (2-second resolution) within the corresponding sampling interval were initially extracted. A data preprocessing step was then applied, including (i) removal of the initial sensor stabilization period, (ii) exclusion of non-physical or invalid readings, and (iii) elimination of sporadic artifacts and outliers. Consequently, *N* denotes the number of valid 2-second sensor observations retained after preprocessing rather than the total number of raw measurements within the sampling interval. Table 2 summarizes the sampling collections for each microbial class.

Given that the sensor’s data did not meet normality or homoscedasticity assumptions, only non-parametric statistical methods were applied. Inter-group differences across all sensing variables were statistically evaluated using the Kruskal–Wallis H test, complemented by pairwise Mann–Whitney U comparisons to characterize specific contrasts between the control and cultured conditions. Effect size metrics (Cliff’s δ), median shifts (Δ-median), and discretized significance levels were computed to quantify both the magnitude and direction of distributional changes across experimental classes. All statistical analyses were performed using Python (version 3.9.21; Python Software Foundation, Wilmington, DE, USA). The code supporting this work is publicly available on GitHub (version 2.47.0) at [38].

## 3. Results

### 3.1. The Prototype Architecture

**Figure 5:**
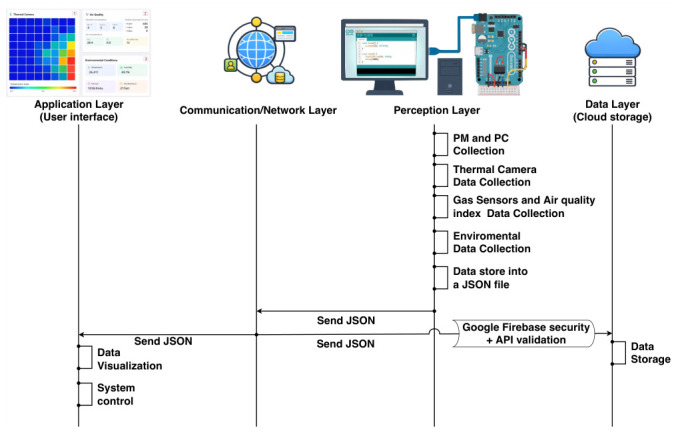
Layered architecture of the prototype, showing the interaction between the perception, communication/network, data and application layers.

The proposed prototype follows a layered architecture, where each component performs a specific role within the data pipeline illustrated in Figure 5. The perception layer acquires raw data from multiple sources—including PM sensors, gas sensors, environmental sensors, and a thermal camera—which are locally processed and structured into JSON format. The communication/network layer ensures reliable data transmission to external services, while the data layer manages cloud storage and security through API validation and remote database integration (e.g., Firebase). Finally, the application layer enables real-time data visualization and system control. This architecture allowed us to obtain the experimental results necessary to evaluate the performance of data acquisition, transmission and visualization across the system.

### 3.2. Biological Ground-Truth Validation

Biological load was quantified using the impaction method with an SAS sampler. For each sampling cycle, the device aspirated 1000 L of air, ensuring a standardized sampling volume across the assays. Under these conditions, the number of colonies counted on each Petri dish corresponded directly to the airborne microbial concentration, expressed in CFU/m^3^. Table 3 summarizes the colony counts obtained from the four independent samples (A, B, C and D) collected for each assay.

### 3.3. Prototype Hardware Validation via Statistical Tests

Statistical analyses were performed to assess the inter-group differences across the 15 recorded time-series variables. Global variability was first evaluated using the non-parametric Kruskal–Wallis H test (Table 4), which is appropriate for non-Gaussian sensor data and unequal variances. All the variables exhibited statistically significant group effects (p<0.05). To enhance interpretability beyond the raw *H* statistic and associated *p*-value, the results were further expressed as $-\log_{10}\left(p\right)$ and discretized significance levels. Significance stars were encoded using standard probability thresholds (*: p<0.05, **: p<0.01, ***: p<0.001, ****: $p<10^{-4}$), providing an ordinal threshold-based summary of evidence strength.

Pairwise analyses were then assessed using the Mann–Whitney U test (Table 5), which likewise confirmed significant differences across all the comparisons (p<0.05). No correction for multiple comparisons was applied because each time-series variable represents an independent sensing feature. Nevertheless, the overall pattern of significance was computed, and it remained consistent when verified under a false discovery rate (FDR) framework (*q*), which was computed only as a robustness check. To quantify the direction and magnitude of between-group shifts, median differences (Δ-median) were computed as Δ=median(GroupA)−median(GroupB), where Δ>0 indicates higher values in Group A (↑) and Δ<0 indicates higher values in Group B (↓). Effect sizes were estimated using Cliff’s δ, a non-parametric probabilistic index ranging from −1 to +1, with values near zero reflecting minimal separability between groups and larger absolute values indicating stronger effects. Similar to the previous analysis, $-\log_{10}\left(p\right)$ values and significance stars were also reported.

### 3.4. Prototype Hardware Validation via Machine Learning Tools

To assess the pairwise and all-vs-all classification performance across the study groups, we evaluated three scikit-learn ML classifiers [39] (Table 6) using a repeated stratified 80/20 train–test split over 50 distinct random seeds. This strategy approximates the robustness of repeated cross-validation as each model is assessed on 50 independent test sets.

For each random seed, an identical preprocessing and training pipeline was fitted exclusively on the training data, ensuring strict isolation from data leakage. The pipeline comprised three sequential steps: (i) z-score normalization of all time-series features; (ii) feature selection using an ANOVA F-value, where features were ranked by discriminatory power and subsets ranging from one to 15 features were evaluated to identify the optimal configuration; and (iii) model training on the transformed feature space.

The resulting trained models were evaluated on the corresponding held-out test sets, which were never used during feature selection, using the following metrics [40,41]:

- Accuracy (%), which represents the proportion of correctly classified samples among all samples and is defined as [42]
  (1) $$Accuracy=\frac{TP+TN}{TP+TN+FP+FN}\times 100\%$$
  where TP, TN, FP, and FN denote the numbers of true positives, true negatives, false positives, and false negatives, respectively [43].
- Precision (%) (also referred to as the positive predictive value), which measures the proportion of correctly predicted positive samples among all samples predicted as positive [44]:
  (2) $$Precision=\frac{TP}{TP+FP}\times 100\%$$
- Recall (%) (also known as sensitivity or true positive rate), which corresponds to the proportion of correctly predicted positive samples relative to the total number of actual positive samples [44]:
  (3) $$Recall=\frac{TP}{TP+FN}\times 100\%$$
- F1-score, which is defined as the harmonic mean of *precision* and *recall*, providing a balanced measure of classification performance [45]:
  (4) $$F1\text{-}Score=\frac{2\times Precision\times Recall}{Precision+Recall}$$
- Area under the receiver operating characteristic curve (*AUC*), which evaluates the model’s ability to discriminate between classes by summarizing the trade-off between the true positive rate and the false positive rate across different decision thresholds. *AUC* values range from 0 to 1, where 1 indicates perfect discrimination and 0.5 corresponds to random classification [46].

All the reported performance metrics correspond to averages across the 50 random-state runs, providing stable and reliable estimates of classification performance. The best results for each comparison across the 50 random-state runs (mean ± standard deviation) are presented in Table 7.

## 4. Discussion

### 4.1. Statistical Tests for Sensor Validation

The results presented in Table 3 confirm that the volumetric impaction method using the SAS sampler provided a robust and reproducible biological ground truth for sensor validation. The measured airborne microbial loads ranged from baseline laboratory levels of approximately $6\;\mathrm{CFU}/m^{3}$ to peak dispersion events of about $165\;\mathrm{CFU}/m^{3}$, establishing a controlled and well-defined gradient of biological exposure. The slight reduction observed in mature cultures is consistent with the expected decrease in spore detachment due to dense mycelial mat formation. It is important to note that these concentrations remain well below commonly referenced guideline thresholds. The WHO expert group suggests that total microbial loads in indoor environments should not exceed 1000 CFU/m^3^, while more conservative recommendations propose limits between 300 CFU/m^3^ and 750 CFU/m^3^ [14]. However, the use of lower concentrations allowed testing under controlled indoor conditions, supporting an initial validation of the system in realistic baseline scenarios. Despite this, these conditions are not sufficient to test the prototype’s limits. Higher microbial loads could introduce additional complexities, such as sensor saturation or less stable sensor behavior. Therefore, further experiments under elevated concentrations and more stressful environmental conditions are necessary to comprehensively evaluate the robustness, sensitivity and scalability of the proposed system.

The Kruskal–Wallis H test (Table 4) revealed statistically significant differences across all the experimental classes for every measured variable (p<0.001 in all cases). The H statistics ranged from 415.09 for NO_2_ to 1716.23 for pressure, indicating substantial distributional shifts caused by the experimental conditions. The corresponding $-\log_{10}\left(p\right)$ values further highlight the magnitude of these effects, with several variables exceeding 300, consistent with *p*-values below $10^{-300}$. All the variables reached four-star significance (****), demonstrating that the class-dependent differences were not only statistically robust but also practically meaningful. Variables such as PM_1.0_, pressure and humidity showed the strongest effects, whereas NO_2_ displayed comparatively lower (yet still highly significant) statistics.

Pairwise comparisons conducted with the Mann–Whitney U test and global FDR correction (Table 5) confirmed significant differences between classes for most variables (q<0.05). The gas resistance, humidity, pressure and particulate metrics yielded the largest effect sizes, with Cliff’s |δ| values frequently approaching 1, indicating near complete distributional separation. Gas resistance was consistently higher in the second culture relative to all the other classes, while the first culture tended to show lower values, suggesting distinct gas emission or absorption dynamics. Humidity also varied substantially: the third culture exhibited the highest values, whereas the control and first cultures presented lower medians, consistent with differing biological activity or chamber microenvironments. The PM (PM_1.0_, PM_2.5_, and PM_10.0_) and PC metrics (PC_>0.3_ to PC_>5.0_) showed large effect sizes across multiple comparisons, confirming pronounced shifts in particulate behavior between classes. The second culture recorded the highest particulate levels, while the first culture showed the lowest, with the control generally presenting intermediate patterns. The differences between the third culture and the control were smaller, yielding weak or non-significant contrasts in several cases. These findings confirm that the particle concentrations inside the chamber varied meaningfully across the experimental conditions and that relative particle size distributions offer more discriminatory power than absolute PM levels alone.

The gas-related variables also demonstrated clear differences between groups. NO_2_ displayed strong class-dependent variation, with the effect direction depending on the specific comparison. O_3_ showed significant changes except between the second culture and the control, where no statistical difference was detected, implying similar oxidative profiles under those conditions. The AQI patterns followed the combined behavior of particulate and gaseous metrics, with the first culture consistently presenting higher medians and the control again occupying an intermediate position. Pressure and temperature also differed significantly, although their median shifts were smoother, indicating weaker practical effects despite small p-values.

### 4.2. Machine Learning Sensor Validation

The classification results demonstrate an exceptionally high discriminative capacity across all the binary and multi-class tasks. In all cases, the evaluated models achieved near-perfect or perfect performance, with *accuracy*, *AUC*, *F1-score*, *precision* and *recall* consistently reaching 100% or values very close to it. This behavior is particularly evident for the ExTrees classifier, which consistently achieved perfect classification across all the binary comparisons and feature configurations, even when evaluated over 50 different training and testing splits. In several binary tasks, perfect classification was achieved using a single dominant feature (pressure), suggesting that certain variables alone carry sufficient discriminative information to separate the classes. In more complex comparisons, additional environmental and sensor-derived features (e.g., gas resistance, temperature, AQI-related measures, O_3_, and NO_2_) were also included yet still yielded perfect separation, indicating both a high degree of separability between the different cultures and the recurrence of specific features among the most relevant predictors across multiple classification tasks. Across the 21 evaluated models, the feature selection analysis revealed clear dominance of a small subset of variables. In particular, pressure was selected in all the models (100%), making it by far the most recurrent feature. This was followed by gas resistance, AQI and temperature, each appearing in seven out of the 21 models (33.3%), while AQI, O_3_, PM_1.0_ and PC_>5.0_ appeared in five out of the 21 models (23.8%). The high recurrence of these features across independent models and classification tasks suggests that they capture consistent and robust differences between the analyzed conditions and may therefore be considered strong candidate biomarkers for distinguishing the tested conditions.

Recent studies on indirect fungal contamination detection have demonstrated the feasibility of using environmental sensors, electronic noses and ML techniques to overcome the limitations of conventional laboratory-based methods, which are slow, labor-intensive and unable to capture non-viable spores. VOC-based sensing systems, MOX sensor arrays and deep learning architectures typically report *accuracy* values ranging from approximately 87% to 96%. However, these approaches commonly rely on a single sensing modality, often require proprietary or laboratory-intensive infrastructure, and frequently lack direct quantitative microbiological validation, limiting the interpretation of performance metrics in terms of actual airborne fungal load. In contrast, the system proposed in this work not only confirms the effectiveness of indirect detection strategies but also demonstrates improved performance compared to the current state of the art. The classification results show an exceptionally high discriminative capacity, with *accuracy*, *AUC*, *F1-score*, *precision* and *recall* consistently reaching values of 100% or very close to it across all the binary and multi-class tasks and remaining stable over multiple training and testing splits. This performance exceeds that reported by most VOC-based and electronic-nose systems, including those relying on advanced deep learning models. Furthermore, while many existing approaches require high-dimensional feature spaces and complex sensing configurations, the present results show that a very limited subset of variables is sufficient to achieve perfect discrimination. In particular, pressure emerged as the dominant feature, being selected in 100% of the evaluated models and, in several cases, enabling perfect classification on its own. The consistent recurrence of pressure, gas resistance, AQI and temperature suggests the presence of robust and stable indirect environmental biomarkers. A key distinguishing aspect of this work is the integration of rigorous quantitative microbiological validation based on volumetric inertial-impaction sampling, providing direct ground truth in terms of airborne fungal concentration (CFU/m^3^). This level of biological validation is largely absent from most ML-based systems reported to date, where high performance metrics may not accurately reflect real bioaerosol levels. Finally, the proposed system was developed following a fully open-source philosophy, encompassing hardware architecture and data acquisition procedure pipelines. Unlike most previously reported solutions, which are proprietary or only partially disclosed, this open-source approach enhances reproducibility, independent validation and extensibility. Taken together, these aspects position the proposed system as a solution that not only surpasses the state of the art in performance but does so with greater simplicity, transparency and experimental rigor.

The statistical and ML results are generally consistent and complement each other in showing separation between the experimental conditions. The statistical tests show that all the measured variables exhibit significant differences across the experimental groups, with pressure, PM_1.0_, PM_2.5_, PM_10.0_, PC_>0.3_, gas resistance and humidity consistently presenting large effect sizes and significant differences across multiple pairwise comparisons. In contrast, variables such as NO_2_ display comparatively weaker or more context-dependent differences despite remaining statistically significant at the global level. These statistical findings are generally reflected in the ML results. However, while all the variables were statistically significant, the ML results indicate that only a subset of features is sufficient to achieve optimal classification. In several cases, perfect classification was achieved using a single feature, most notably pressure, which aligns with its extremely high H statistic and consistently large effect sizes across pairwise comparisons. Pressure also presents the highest $-\log_{10}\left(p\right)$ values among all the variables, further reinforcing its dominant statistical relevance and helping to explain its consistent selection across all the ML models. A similar relationship can be observed for other variables, such as PM_1.0_, gas resistance, temperature and humidity, although with lower consistency. However, variables such as PC_>0.3_ or PM_2.5_, despite showing strong statistical significance, are only selected in specific scenarios (e.g., the “all vs all” task), suggesting that their discriminative information may be redundant when combined with other features or less important for simpler binary separations.

## 5. Conclusions

The development of real-time multimodal sensing systems is crucial for the early detection of biological contamination in indoor environments, enabling timely interventions before harmful exposure occurs. This study addresses a central challenge in preventive conservation and indoor public health by introducing a scalable, affordable and real-time system that is capable of detecting biological contamination in indoor environments. By combining multimodal sensing with rigorous statistical validation and ML, the study demonstrates that indirect environmental measurements can reliably act as proxies for fungal presence when analyzed with appropriate methodologies.

The results show that the sensor array successfully distinguished the experimental classes, with all the measured variables showing statistically significant differences. Particle concentrations, gaseous indicators and humidity varied markedly with fungal activity, with particulate metrics and MOX gas resistance producing the strongest contrasts. In addition to statistical analysis, the classification results confirmed the strong discriminative capability of the proposed system, with near-perfect or perfect performance across all the tested scenarios. The consistent results obtained across different models and configurations indicate a high degree of separability between the different tested cultures and support the robustness of the selected features. Pressure emerged as the most informative variable, consistently dominating in both statistical and ML analyses. In contrast, variables such as NO_2_, PC_>0.3_ and PM_2.5_ exhibited weaker contributions despite remaining statistically significant. Overall, these results suggest that changes in key environmental signals can be used as early indicators of biological activity and potential contamination.

Furthermore, the system demonstrated sufficient sensitivity to detect airborne fungal spore presence at low concentration levels within the tested range. By allowing rapid real-time detection of biological contamination, this system may identify threats earlier than traditional culture-based methods, reducing health risks, preventing material degradation and supporting proactive interventions in conservation, clinical and occupational environments.

Future work should explore ML approaches for on-device anomaly detection, allowing deviations from baseline behavior to trigger alerts without pathogen-specific training. Deep learning models may also improve the capture of nonlinear interactions in multisensor datasets, especially when integrated with IoT infrastructures for real-time calibration, drift correction and bioaerosol forecasting. Hardware refinements, such as differential indoor–outdoor sensing, may help to address limitations linked to particulate matter variability. Long-term deployments will require systematic evaluation of drift, chemical aging and cross-sensitivities in MOX sensors to support adaptive calibration strategies. Real-world validation in museums, archives, hospitals and other sensitive environments is essential to assess robustness under dynamic operating conditions influenced by the environment, human presence and seasonal fluctuations. Finally, expanding the biological scope to include additional fungal species, such as *Aspergillus fumigatus*, *Cladosporium herbarum* and *Stachybotrys chartarum*, is essential. These species differ substantially in aerodynamic behavior, aggregation patterns and volatile emission profiles. Their inclusion will broaden the applicability of the sensing system and impose a more rigorous stress test on the architecture. Such investigations may require targeted adjustments to the prototype, including refined airflow control, optimized impactor efficiency, recalibration of MOX responses and adjusted optical thresholds, ensuring robust performance across diverse bioaerosol types.

## Figures and Tables

**Table 1:** Detection characteristics and metrological performance of sensors used in the experimental setup.

Sensor	Measured Variable(s)	Detection and Sensitivity Characteristics	Resolution/Output and Performance	Minimum Limit of Detection
PMSA003I (Plantower) [34]	PM_1.0_, PM_2.5_, PM_10.0_	Laser scattering optical sensor. Detects particles ≥0.3 µm (50% CE; ≥0.5 µm at ∼98% CE). Sample volume ≈0.1 L.	1 µg·m^−3^ step. Max. RE ±10% (100–500 µg·m^−3^) or ±10 µg·m^−3^ (0–100 µg·m^−3^). RT: <1 s (single), ≤10 s (stabilized).	Particle diameter: 0.3 µm
AMG8833 (Panasonic Grid-EYE) [35]	IR (8 × 8)	Thermal microbolometer array. HGM, FOV 60°. NETD ≈0.05 K @1 Hz (0.16 K @10 Hz).	12-bit output (0.25 K LSB). AA ±2.5 K (0–80 °C). FR: 1 or 10 Hz.	Temperature difference: 0.05 K
BME680 (Bosch) [36]	T, RH, P, IAQ, eCO_2_	4-in-1 sensor: MOX gas sensor + T/RH/P. VOC-sensitive (ppb–ppm). Algorithm-based IAQ output (BSEC).	T: ±1.0 °C; RH: ±3%; P: ±1 hPa. Gas: IAQ (0–500), eCO_2_/TVOC (algorithm-derived, relative).	Gas resistance change: 0.05%
Nicla Sense Env [25]	T, RH, O_3_, TVOC, eCO_2_, NO_2_	HS4001 (T/RH) + ZMOD4410 (IAQ) + ZMOD4510 (outdoor AQ). MOX sensors with onboard ML processing.	T: ±0.2 °C; RH: ±1.5% (typ.). Gas: AQ indices and estimated concentrations (firmware-derived, no absolute accuracy).	Temperature: −40 °C; NO_2_ and O_3_: 20 ppb; TVOC: 1 µg/m^3^

Abbreviations: AA: absolute accuracy; AQ: air quality; BSEC: Bosch Software Environmental Cluster; CE: counting efficiency; eCO_2_: equivalent carbon dioxide concentration; FOV: field of view; FR: frame rate; HGM: high-gain mode; IAQ: indoor air quality (index); IR: infrared; LSB: least significant bit; ML: machine learning; MOX: metal–oxide (gas sensor); NETD: noise-equivalent temperature difference; P: barometric pressure; PM: particulate matter; RE: repeatability error; RH: relative humidity; RT: response time; T: temperature; TVOC: total volatile organic compounds; VOC: volatile organic compounds.

**Table 2:** Summary of sampling collections for each microbial class, including dates, times and number of collected samples (N).

Class	Date	Start Time	End Time	N
1st culture	22/09/2025	7:58	8:16	420
2nd culture	14/10/2025	10:58	11:43	549
3rd culture	22/10/2025	10:50	11:16	513
control	22/10/2025	11:22	11:43	507

**Table 3:** Quantification of biological load (CFU/m^3^) across experimental assays.

Experimental Phase	Sample A	Sample B	Sample C	Sample D	Mean ($\bar{x}$)
**Control**	11	5	4	5	**6.25**
**Assay 1 (1st Culture)**	111	104	109	118	**110.50**
**Assay 2 (2nd Culture)**	173	163	150	175	**165.25**
**Assay 3 (3rd Culture)**	127	130	134	132	**130.75**

**Table 4:** Kruskal–Wallis H-test results with $-\log_{10}\left(p\right)$ and significance star levels.

Variable	H	*p*	$-\log_{10}\left(p\right)$	Stars
AQI	1070.5245	$9.03\times 10^{-232}$	231.04	****
Gas Resistance	1533.6529	<$1\times 10^{-300}$	>300	****
Humidity	1641.5978	<$1\times 10^{-300}$	>300	****
NO_2_	415.0858	$1.19\times 10^{-89}$	88.92	****
O_3_	839.1474	$1.40\times 10^{-181}$	180.85	****
PC_>0.3_	1573.3739	<$1\times 10^{-300}$	>300	****
PC_>0.5_	1573.6878	<$1\times 10^{-300}$	>300	****
PC_>1.0_	1535.2706	<$1\times 10^{-300}$	>300	****
PC_>2.5_	772.2167	$4.59\times 10^{-167}$	166.34	****
PC_>5.0_	521.7458	$9.24\times 10^{-113}$	112.03	****
PM_1.0_	1671.9412	<$1\times 10^{-300}$	>300	****
PM_2.5_	1630.0934	<$1\times 10^{-300}$	>300	****
PM_10.0_	1640.2105	<$1\times 10^{-300}$	>300	****
Pressure	1716.2255	<$1\times 10^{-300}$	>300	****
Temperature	1364.3607	$1.59\times 10^{-295}$	294.80	****

Note: ****: $p<10^{-4}$.

**Table 5:** Mann–Whitney U-test results for all pairwise comparisons.

Variable	Comparison	U	*p*	*d_median_*	δ	*q*	$-\log_{10}\left(p\right)$	Dir	Stars
AQI	1st culture vs. 3rd culture	215,460	$1.8214\times 10^{-156}$	17	−1	$6.3048\times 10^{-156}$	155.7396	↑	****
AQI	1st culture vs. 2 culture	225,669	$1.0505\times 10^{-146}$	16	−0.9574	$2.4880\times 10^{-146}$	145.9786	↑	****
AQI	1st culture vs. control	206,554.5	$3.7692\times 10^{-135}$	11	−0.9400	$7.0672\times 10^{-135}$	134.4238	↑	****
AQI	3rd culture vs. control	65,872	$2.2611\times 10^{-45}$	−6	0.4935	$3.1307\times 10^{-45}$	44.6457	↓	****
AQI	2nd culture vs. 3rd culture	168,794	$3.0010\times 10^{-9}$	1	−0.1987	$3.5076\times 10^{-9}$	8.5227	↑	****
AQI	2nd culture vs. control	113,499	$1.0614\times 10^{-7}$	−5	0.1845	$1.2247\times 10^{-7}$	6.9741	↓	****
Gas Resistance	2nd culture vs. control	278,343	$7.9203\times 10^{-174}$	90,000	−1	$5.4833\times 10^{-173}$	173.1013	↑	****
Gas Resistance	1st culture vs. 2 culture	680.5	$2.7126\times 10^{-155}$	−47,300	0.9941	$9.0421\times 10^{-155}$	154.5666	↓	****
Gas Resistance	1st culture vs. control	212,595	$9.1488\times 10^{-151}$	42,700	−0.9968	$2.4217\times 10^{-150}$	150.0386	↑	****
Gas Resistance	3rd culture vs. control	250,394.5	$2.3213\times 10^{-144}$	48,700	−0.9254	$4.8584\times 10^{-144}$	143.6343	↑	****
Gas Resistance	2nd culture vs. 3rd culture	245,668	$7.7727\times 10^{-98}$	41,300	−0.7446	$1.2719\times 10^{-97}$	97.1094	↑	****
Gas Resistance	1st culture vs. 3rd culture	76,854.5	$4.7101\times 10^{-14}$	−6000	0.2866	$5.6521\times 10^{-14}$	13.3270	↓	****
Humidity	2nd culture vs. 3rd culture	0	$4.6850\times 10^{-175}$	−17.9	1	$5.2707\times 10^{-174}$	174.3293	↓	****
Humidity	2nd culture vs. control	0	$5.0950\times 10^{-174}$	−15	1	$3.8213\times 10^{-173}$	173.2929	↓	****
Humidity	1st culture vs. 3rd culture	0	$1.1320\times 10^{-152}$	−17.3	1	$3.3960\times 10^{-152}$	151.9462	↓	****
Humidity	1st culture vs. control	0	$6.8102\times 10^{-152}$	−14.4	1	$1.9154\times 10^{-151}$	151.1668	↓	****
Humidity	3rd culture vs. control	242,732	$6.1395\times 10^{-127}$	2.9	−0.8665	$1.1051\times 10^{-126}$	126.2119	↑	****
Humidity	1st culture vs. 2nd culture	116,819.5	0.7230	0.6	−0.0133	0.7311	0.1409	↑	
NO_2_	1st culture vs. 2nd culture	201,329	$1.0373\times 10^{-88}$	21.25	−0.7463	$1.6096\times 10^{-88}$	87.9841	↑	****
NO_2_	2nd culture vs. control	68,526	$5.8332\times 10^{-47}$	−30.3	0.5076	$8.2029\times 10^{-47}$	46.2341	↓	****
NO_2_	3rd culture vs. control	71,429.5	$4.5383\times 10^{-37}$	−32.9	0.4507	$6.1886\times 10^{-37}$	36.3431	↓	****
NO_2_	1st culture vs. control	69,722.5	$1.2071\times 10^{-19}$	−9.05	0.3451	$1.5301\times 10^{-19}$	18.9183	↓	****
NO_2_	1st culture vs. 3rd culture	140,093	$1.6828\times 10^{-15}$	23.85	−0.3004	$2.0466\times 10^{-15}$	14.7740	↑	****
NO_2_	2nd culture vs. 3rd culture	127,259	0.0055	2.6	0.0963	0.0058	2.2580	↑	**
O_3_	1st culture vs. 2nd culture	189,405	$4.1448\times 10^{-105}$	0.1	−0.6429	$7.1737\times 10^{-105}$	104.3825	↑	****
O_3_	1st culture vs. control	174,915	$1.3893\times 10^{-98}$	0.1	−0.6429	$2.3155\times 10^{-98}$	97.8572	↑	****
O_3_	1st culture vs. 3rd culture	161,731	$2.8143\times 10^{-50}$	0.1	−0.5013	$4.0853\times 10^{-50}$	49.5506	↑	****
O_3_	2nd culture vs. 3rd culture	108,976.5	$3.9287\times 10^{-32}$	0	0.2261	$5.2773\times 10^{-32}$	31.4058	↓	****
O_3_	3rd culture vs. control	159,451.5	$6.0614\times 10^{-30}$	0	−0.2261	$8.0224\times 10^{-30}$	29.2174	↓	****
O_3_	2nd culture vs. control	139,171.5	1	0	0	1	0	↓	
PC_>0.3_	2nd culture vs. 3rd culture	277,020	$9.9903\times 10^{-164}$	3843	−0.9672	$4.7322\times 10^{-163}$	163.0004	↑	****
PC_>0.3_	2nd culture vs. control	273,802.5	$8.4988\times 10^{-163}$	3819	−0.9674	$3.4768\times 10^{-162}$	162.0706	↑	****
PC_>0.3_	1st culture vs. 3rd culture	303	$1.0869\times 10^{-151}$	−438	0.9972	$2.9642\times 10^{-151}$	150.9638	↓	****
PC_>0.3_	1st culture vs. 2nd culture	3780	$4.1060\times 10^{-147}$	−4281	0.9672	$9.9876\times 10^{-147}$	146.3866	↓	****
PC_>0.3_	1st culture vs. control	2370.5	$3.8134\times 10^{-145}$	−462	0.9777	$8.3710\times 10^{-145}$	144.4187	↓	****
PC_>0.3_	3rd culture vs. control	114,975	0.0014	−24	0.1159	0.0015	2.8675	↓	**
PC_>0.5_	2nd culture vs. 3rd culture	277,020	$9.9609\times 10^{-164}$	1137	−0.9672	$4.9805\times 10^{-163}$	163.0017	↑	****
PC_>0.5_	2nd culture vs. control	273,802.5	$8.4735\times 10^{-163}$	1130	−0.9674	$3.6315\times 10^{-162}$	162.0719	↑	****
PC_>0.5_	1st culture vs. 3rd culture	190	$5.2088\times 10^{-152}$	−137	0.9982	$1.5122\times 10^{-151}$	151.2833	↓	****
PC_>0.5_	1st culture vs. 2nd culture	3780	$4.0841\times 10^{-147}$	−1274	0.9672	$1.0210\times 10^{-146}$	146.3889	↓	****
PC_>0.5_	1st culture vs. control	2316	$2.6733\times 10^{-145}$	−144	0.9782	$6.0150\times 10^{-145}$	144.5729	↓	****
PC_>0.5_	3rd culture vs. control	116,285.5	0.0034	−7	0.1058	0.0037	2.4631	↓	**
PC_>1.0_	2nd culture vs. 3rd culture	277,020	$8.9510\times 10^{-164}$	172	−0.9672	$4.7387\times 10^{-163}$	163.0481	↑	****
PC_>1.0_	2nd culture vs. control	273,802.5	$7.7500\times 10^{-163}$	168	−0.9674	$3.4875\times 10^{-162}$	162.1107	↑	****
PC_>1.0_	1st culture vs. 2nd culture	3739.5	$2.1738\times 10^{-147}$	−206	0.9676	$5.5897\times 10^{-147}$	146.6628	↓	****
PC_>1.0_	1st culture vs. 3rd culture	5678.5	$2.6222\times 10^{-137}$	−34	0.9473	$5.0213\times 10^{-137}$	136.5813	↓	****
PC_>1.0_	1st culture vs. control	6561	$4.6279\times 10^{-134}$	−38	0.9384	$8.5002\times 10^{-134}$	133.3346	↓	****
PC_>1.0_	3rd culture vs. control	112,145	0.0001	−4	0.1376	0.0002	3.8541	↓	***
PC_>2.5_	1st culture vs. 2nd culture	15,697.5	$1.0863\times 10^{-119}$	−10	0.8638	$1.9171\times 10^{-119}$	118.9640	↓	****
PC_>2.5_	1st culture vs. control	20,626.5	$6.4378\times 10^{-102}$	−6	0.8063	$1.0932\times 10^{-101}$	101.1913	↓	****
PC_>2.5_	1st culture vs. 3rd culture	24,768.5	$1.2086\times 10^{-93}$	−6	0.7701	$1.9424\times 10^{-93}$	92.9177	↓	****
PC_>2.5_	2nd culture vs. 3rd culture	195,579	$3.0364\times 10^{-28}$	4	−0.3889	$3.9605\times 10^{-28}$	27.5176	↑	****
PC_>2.5_	2nd culture vs. control	189,212	$3.00237\times 10^{-24}$	4	−0.3596	$3.8602\times 10^{-24}$	23.5225	↑	****
PC_>2.5_	3rd culture vs. control	126,252	0.4158	0	0.0292	0.4252	0.3811	↓	
PC_>5.0_	1st culture vs. control	28,074	$3.8639\times 10^{-88}$	−4	0.7363	$5.8940\times 10^{-88}$	87.4130	↓	****
PC_>5.0_	1 culture vs. 3rd culture	29,845	$3.0186\times 10^{-85}$	−4	0.7230	$4.5279\times 10^{-85}$	84.5202	↓	****
PC_>5.0_	1 culture vs. 2 culture	55,001	$4.0832\times 10^{-48}$	−4	0.5229	$5.8331\times 10^{-48}$	47.3890	↓	****
PC_>5.0_	2 culture vs. 3rd culture	101,845	$1.4399\times 10^{-15}$	0	0.2768	$1.7752\times 10^{-15}$	14.8417	↓	****
PC_>5.0_	2 culture vs. control	104,142.5	$3.6704\times 10^{-13}$	0	0.2517	$4.3465\times 10^{-13}$	12.4353	↓	****
PC_>5.0_	3rd culture vs. control	135,608	0.2256	0	−0.0428	0.2334	0.6467	↓	
PM_1.0_	2nd culture vs. 3rd culture	281,637	$9.3348\times 10^{-177}$	28	−1	$2.8005\times 10^{-175}$	176.0299	↑	****
PM_1.0_	2nd culture vs. control	278,333.5	$1.6851\times 10^{-175}$	28	−0.9999	$3.0331\times 10^{-174}$	174.7734	↑	****
PM_1.0_	1st culture vs. 2nd culture	0	$1.6895\times 10^{-159}$	−32	1	$6.6110\times 10^{-159}$	158.7722	↓	****
PM_1.0_	1st culture vs. 3rd culture	1391.5	$8.6914\times 10^{-153}$	−4	0.9871	$2.7937\times 10^{-152}$	152.0609	↓	****
PM_1.0_	1st culture vs. control	1015.5	$8.9657\times 10^{-153}$	−4	0.9905	$2.7825\times 10^{-152}$	152.0474	↓	****
PM_1.0_	3rd culture vs. control	112,169	$8.1113\times 10^{-5}$	0	0.1375	$9.1253\times 10^{-5}$	4.0909	↓	****
PM_2.5_	2nd culture vs. 3rd culture	281,637	$1.1124\times 10^{-175}$	38	−1	$2.5028\times 10^{-174}$	174.9538	↑	****
PM_2.5_	2nd culture vs. control	278,342	$2.2375\times 10^{-174}$	37	−0.1000	$2.0138\times 10^{-173}$	173.6502	↑	****
PM_2.5_	1st culture vs. 2nd culture	0	$1.3080\times 10^{-158}$	−42	1	$4.9049\times 10^{-158}$	157.8834	↓	****
PM_2.5_	1st culture vs. control	4720	$1.1603\times 10^{-140}$	−5	0.9557	$2.3734\times 10^{-140}$	139.9354	↓	****
PM_2.5_	1st culture vs. 3rd culture	5280	$2.5059\times 10^{-140}$	−4	0.9510	$5.0117\times 10^{-140}$	139.6010	↓	****
PM_2.5_	3rd culture vs. control	108,301.5	$2.8629\times 10^{-6}$	−1	0.1672	$3.2615\times 10^{-6}$	5.5432	↓	****
PM_10.0_	2nd culture vs. 3rd culture	281,627.5	$4.0927\times 10^{-175}$	36	−0.9999	$5.2620\times 10^{-174}$	174.3880	↑	****
PM_10.0_	2nd culture vs. control	278,329.5	$4.4377\times 10^{-174}$	36	−0.9999	$3.6309\times 10^{-173}$	173.3528	↑	****
PM_10.0_	1st culture vs. 2nd culture	0	$7.8445\times 10^{-158}$	−44	1	$2.8240\times 10^{-157}$	157.1054	↓	****
PM_10.0_	1st culture vs. control	2220	$1.9507\times 10^{-146}$	−8	0.9791	$4.5016\times 10^{-146}$	145.7098	↓	****
PM_10.0_	1st culture vs. 3rd culture	3131	$9.5391\times 10^{-145}$	−8	0.9709	$2.0441\times 10^{-144}$	144.0205	↓	****
PM_10.0_	3rd culture vs. control	120,722	$4.6565\times 10^{-2}$	0	0.0717	$4.8730\times 10^{-2}$	1.3319	↓	*
Pressure	2nd culture vs. control	278,343	$4.1719\times 10^{-181}$	230	−1	$3.7547\times 10^{-179}$	180.3797	↑	****
Pressure	2nd culture vs. 3rd culture	281,637	$1.1085\times 10^{-179}$	220	−1	$4.9884\times 10^{-178}$	178.9552	↑	****
Pressure	1st culture vs. control	212,940	$2.5732\times 10^{-175}$	720	−1	$3.8597\times 10^{-174}$	174.5895	↑	****
Pressure	1st culture vs. 2nd culture	230,580	$1.2390\times 10^{-173}$	490	−1	$7.9648\times 10^{-173}$	172.9069	↑	****
Pressure	1st culture vs. 3rd culture	215,460	$5.6240\times 10^{-172}$	710	−1	$3.3744\times 10^{-171}$	171.2500	↑	****
Pressure	3rd culture vs. control	145,568	$3.4309\times 10^{-4}$	10	−0.1194	$3.7656\times 10^{-4}$	3.4646	↑	***
Temperature	2nd culture vs. control	278,343	$5.6234\times 10^{-175}$	1.5	−1	$5.6234\times 10^{-174}$	174.2500	↑	****
Temperature	2nd culture vs. 3rd culture	278,833.5	$2.8715\times 10^{-169}$	2.3	−0.9801	$1.6152\times 10^{-168}$	168.5419	↑	****
Temperature	1st culture vs. 2nd culture	7826	$1.5500\times 10^{-137}$	−2.5	0.9321	$3.0326\times 10^{-137}$	136.8097	↓	****
Temperature	3rd culture vs. control	35,739.5	$3.2658\times 10^{-90}$	−0.8	0.7252	$5.1565\times 10^{-90}$	89.4860	↓	****
Temperature	1st culture vs. control	41,651	$9.0297\times 10^{-58}$	−1	0.6088	$1.3323\times 10^{-57}$	57.0443	↓	****
Temperature	1st culture vs. 3rd culture	71,330.5	$3.7221\times 10^{-19}$	−0.2	0.3379	$4.6527\;\times \;10^{-19}$	18.4292	↓	****

Note: ↑: higher values in Group A, ↓: higher values in Group B; *: *p* < 0.05, **: *p* < 0.01, ***: *p* < 0.001, ****: *p* < 10^−4^.

**Table 6:** Configurations of the scikit-learn classifiers used in this study.

Classifier (Scikit-Learn Class)	Hyperparameters
Extra Trees Classifier (ExTrees)	(n_estimators = 100, criterion = “gini”, max_depth = none, min_samples_split = 2, random_state = 42) + default
Support vector machine (SVM)	(kernel = “rbf”, C = 1.0, γ = “scale”, probability = false) + default
Stochastic gradient descent (SGD)	(loss = “hinge”, penalty = “l2”, alpha = 0.0001, max_iter = 1000, tol = 1e-3) + default

**Table 7:** Classification performance of the tested cultures.

Comparison	Classifier	# Features	Features Selected	*Accuracy* (%)	*AUC*	*F1-Score*	*Precision* (%)	*Recall* (%)
control vs. 1st culture	ExTrees	1	P	100.0 ± 0.0	1.00 ± 0.00	1.00 ± 0.00	100.0 ± 0.00	100.0 ± 0.00
	SVM	1	P	100.0 ± 0.0	1.00 ± 0.00	1.00 ± 0.00	100.0 ± 0.0	100.0 ± 0.0
	SGD	1	P	100.0 ± 0.0	1.00 ± 0.0	1.00 ± 0.00	100.0 ± 0.0	100.0 ± 0.0
control vs. 2nd culture	ExTrees	2	P, PC_>2.5_	100.0 ± 0.0	1.00 ± 0.0	1.0 ± 0.0	100.0 ± 0.0	100.0 ± 0.0
	SVM	4	P, PC_>2.5_, PC_>5.0_, PM_>1.0_	100.0 ± 0.0	1.0 ± 0.0	1.0 ± 0.0	100.0 ± 0.0	100.0 ± 0.0
	SGD	1	P	100.0 ± 0.0	1.0 ± 0.0	1.0 ± 0.0	100.0 ± 0.0	100.0 ± 0.0
control vs. 3rd culture	ExTrees	5	H, GR, T, AQI, O_3_	100.0 ± 0.0	1.0 ± 0.0	1.0 ± 0.0	100.0 ± 0.0	100.0 ± 0.0
	SVM	6	H, GR, T, AQI, O_3_, NO_2_	100.0 ± 0.0	1.0 ± 0.0	1.0 ± 0.0	100.0 ± 0.0	100.0 ± 0.0
	SGD	6	H, GR, T, AQI, O_3_, NO_2_	99.97 ± 0.12	0.9997 ± 0.0012	0.9994 ± 0.0023	99.97 ± 0.12	100.0 ± 0.0
1st culture vs. 2nd culture	ExTrees	1	P	100.0 ± 0.0	1.0 ± 0.0	1.0 ± 0.0	100.0 ± 0.0	100.0 ± 0.0
	SVM	1	P	100.0 ± 0.0	1.0 ± 0.0	1.0 ± 0.0	100.0 ± 0.0	100.0 ± 0.0
	SGD	9	All except H, PC_>1.0_, PC_>2.5_, NO_2_, O_3_, PM_2.5_	100.0 ± 0.0	1.0 ± 0.0	1.0 ± 0.0	100.0 ± 0.0	100.0 ± 0.0
1st culture vs. 3rd culture	ExTrees	1	P	100.0 ± 0.0	1.0 ± 0.0	1.0 ± 0.0	100.0 ± 0.0	100.0 ± 0.0
	SVM	1	P	100.0 ± 0.0	1.0 ± 0.0	1.0 ± 0.0	100.0 ± 0.0	100.0 ± 0.0
	SGD	1	P	100.0 ± 0.0	1.0 ± 0.0	1.0 ± 0.0	100.0 ± 0.0	100.0 ± 0.0
2nd culture vs. 3rd culture	ExTrees	1	P	100.0 ± 0.0	1.0 ± 0.0	1.0 ± 0.0	100.0 ± 0.0	100.0 ± 0.0
	SVM	1	P	100.0 ± 0.0	1.0 ± 0.0	1.0 ± 0.0	100.0 ± 0.0	100.0 ± 0.0
	SGD	2	P, PC_>5.0_	100.0 ± 0.0	1.00 ± 0.0	1.0 ± 0.0	100.0 ± 0.0	100.0 ± 0.0
All vs. All	ExTrees	11	All except PM_2.5_, PC_>1.0_, PC_>2.5_, NO_2_	100.0 ± 0.0	1.0 ± 0.0	1.0 ± 0.0	100.0 ± 0.0	100.0 ± 0.0
	SVM	14	All except PM_2.5_	99.98 ± 0.09	0.9994 ± 0.0027	0.9998 ± 0.0009	99.98 ± 0.08	99.98 ± 0.09
	SGD	14	All except PM_2.5_	99.96 ± 0.12	0.9987 ± 0.0038	0.9996 ± 0.0012	99.96 ± 0.12	99.96 ± 0.12

Abbreviations: GR: gas resistance; H: humidity; PC: particle count; PM: particulate matter; P: pressure; T: temperature.

## Data Availability

The original data presented in the study are openly available on GitHub at https://github.com/MInesBarbosaa/multisensorPrototype (accessed on 31 March 2026).
